# Supplementary material for: Semi-Automated High-Throughput Substrate Screening Assay for Nucleoside Kinases
Source: Int J Mol Sci. 2021 Oct 26;22(21):11558. doi: 10.3390/ijms222111558 (PMC8584170; doi:10.3390/ijms222111558)
Supplement: Supplementary file 1 [file ijms-22-11558-s001.zip › ijms-1421723-supplementary.pdf]

Author contributions 1
Data availability 1
Autoluminescence and luciferase inhibition test with mixed ATP and nucleoside/NMP standards 2
Mixed ATP/AMP standards 3
HPLC chromatograms for *Hs*dCK-catalyzed reactions with natural substrates 4
Comparison of HPLC and the luminescent assay for the determination of ATP consumption and (deoxy)NMP formation in *Hs*dCK-catalysed reactions 5
Semi-automated substrate screening assay for nucleoside kinases. 6
Assay protocol 7
Accuracy of the NK activity assay 8
Activities of four (deoxy)nucleoside kinases with 20 natural and modified nucleoside substrates 9
Typical retention times for the HPLC analysis 10
References 11

Author Contributions (with definitions as recommended by Brand et al.[1])

Conceptualization, K.F.H., M.F., P.N. and A.K.; Formal analysis, K.F.H. and M.F.; Funding acquisition, P.N. and A.K.; Investigation, K.F.H. and M.F.; Methodology, K.F.H. and M.F.; Project administration, K.F.H., M.F., P.N. and A.K.; Resources, P.N. and A.K.; Software, K.F.H. and S.H.; Supervision, P.N. and A.K.; Validation, K.F.H. and M.F.; Visualization, K.F.H., M.F. and A.K.; Writing – original draft, K.F.H and M.F.; Writing – review & editing, K.F.H., M.F., S.H., P.N. and A.K.

All authors have read and agree to the published version of the manuscript.

Data availability

All data depicted visually in the items in the main text as well as in the Supplementary Material is available from an externally hosted Supporting Information.[2]

Table S1. Autoluminescence and luciferase inhibition test with mixed ATP and nucleoside/NMP standards.

Mixed standards		Luminescence [RLU]	Difference	Relative [%]
0.4 mM ATP	-	36550 ± 1086	0	100
0.4 mM ATP	0.4 mM dAdo	33974 ± 81	2576	93
0.4 mM ATP	0.4 mM dCyd	35335 ± 48	1215	97
0.4 mM ATP	0.4 mM dGuo	35713 ± 617	837	98
0.4 mM ATP	0.4 mM Ado	35814 ± 433	735	98
0.4 mM ATP	0.4 mM Cyd	33791 ± 589	2759	92
0.4 mM ATP	0.4 mM Guo	35569 ± 0	981	97
0.4 mM ATP	0.4 mM Thd	36771 ± 766	-221	101
0.4 mM ATP	0.4 mM Urd	35513 ± 397	1037	97
0.4 mM ATP	0.4 mM dAMP	36406 ± 628	144	100
0.4 mM ATP	0.4 mM GMP	36152 ± 395	398	99
0.4 mM ATP	0.4 mM dGMP	36897 ± 497	-348	101
0.4 mM ATP	0.4 mM CMP	36188 ± 242	362	99
0.4 mM ATP	0.4 mM dCMP	36271 ± 365	279	99
0.4 mM ATP	0.4 mM UMP	36299 ± 152	251	99
0.4 mM ATP	0.4 mM TMP	37048 ± 1412	-499	101

Standards were manually prepared in NK reaction buffer. ATP/nucleoside standards were analyzed on one assay plate as triplicates. Mean and standard deviation are indicated.

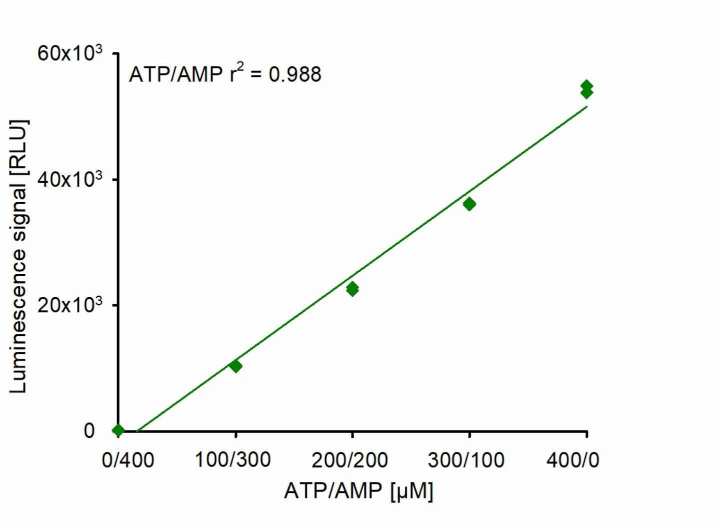

Figure S1. Mixed ATP/AMP standards. Standards were manually prepared in NK reaction buffer as independent duplicates and were also analyzed on different assay plates. Each standard was analyzed by the luminescent assay as duplicate.

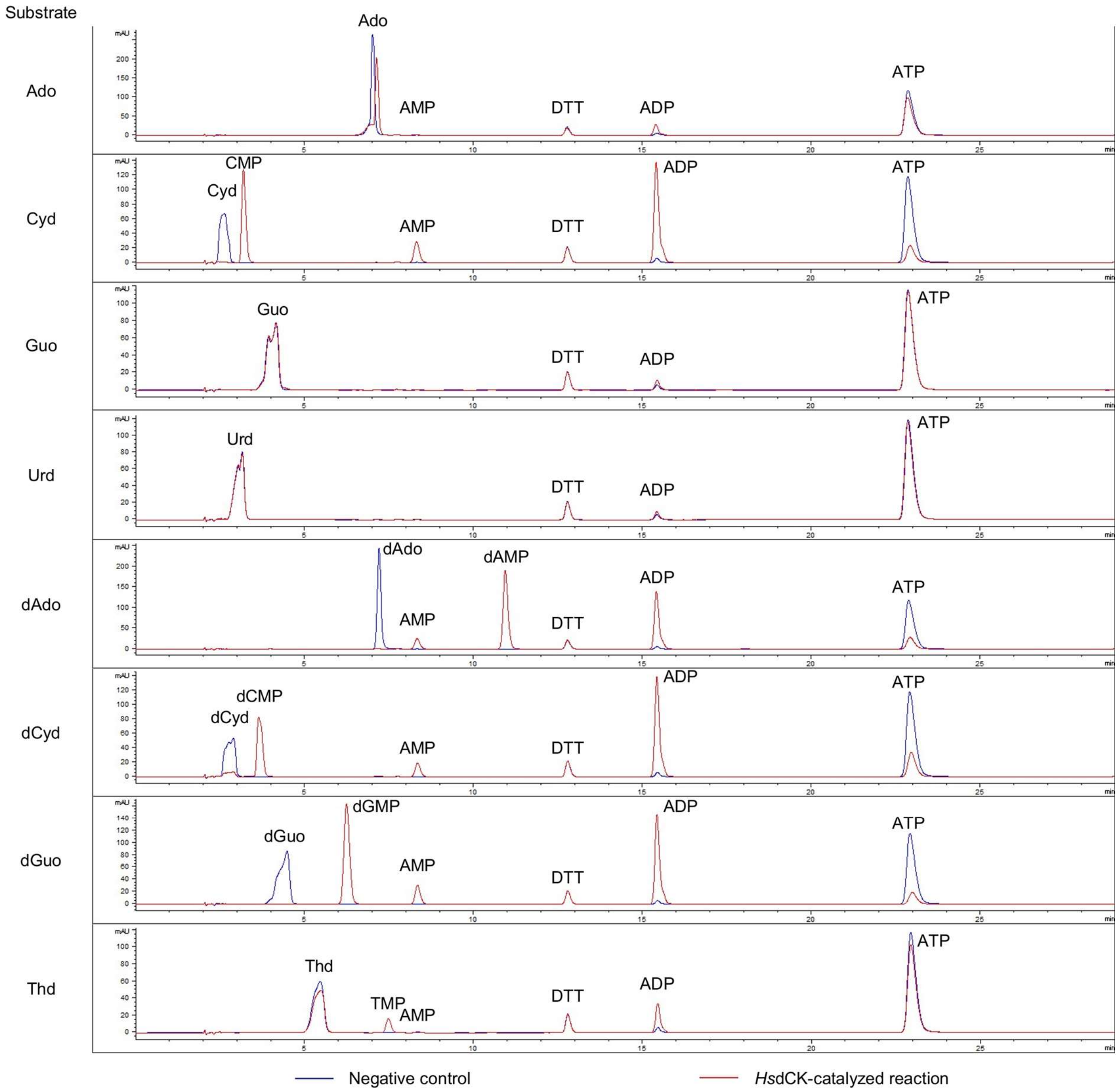

Figure S2. Exemplary HPLC chromatograms for *Hs*dCK-catalyzed reactions with natural substrates.

Table S2. Comparison of HPLC and the luminescent assay for the determination of ATP consumption and (deoxy)NMP formation in *Hs*dCK-catalyzed reactions.

	**Luminescent assay**		**HPLC analysis**		**Difference (Lum – HPLC)**	
	**Consumed ATP [%]**	**(d)NMP [%]**	**Consumed ATP [%]**	**(deoxy)NMP [%]**	**Consumed ATP [%]**	**(deoxy)NMP [%]**
dAdo	80.95 ± 3.95	96.20 ± 3.80	80.12 ± 4.11	99.23 ± 0.16	0.83	-3.03
dCyd	73.62 ± 0.93	88.34 ± 1.11	72.85 ± 0.78	83.98 ± 3.66	0.77	4.36
dGuo	82.32 ± 2.13	98.11 ± 1.89	81.33 ± 2.25	99.26 ± 0.02	0.99	-0.49
Ado	7.41 ± 0.15	8.90 ± 0.17	2.80 ± 0.01	1.37 ± 0.09	4.61	7.53
Cyd	82.64 ± 3.54	97.46 ± 2.54	83.25 ± 3.69	99.10 ± 0.90	-0.60	-1.63
Guo	3.81 ± 2.36	4.57 ± 2.83	0.52 ± 0.52	0.00 ± 0.00	3.29	4.57
Thd	13.15 ± 0.07	15.78 ± 0.08	11.06 ± 1.41	12.90 ± 0.05	2.08	2.88
Urd	3.90 ± 0.36	4.68 ± 0.43	0.00 ± 0.00	0.00 ± 0.00	3.90	4.68

Reactions were manually prepared. Reactions consisting of 70 mM Tris [pH 7.6], 5 mM DTT, 10 mM $MgCl_2$, 0.4 mM ATP, 1/3 mM substrate and 0.0002 U enzyme were prepared in a PCR plate to a final volume of 150 µL. After incubation at 37°C for 19 h, reactions were stopped by heat treatment at 75°C for 10 min. Each reaction was analyzed using the luminescent assay in triplicates. Each reaction was prepared as independent duplicates on different PCR plates and was also analyzed on different assay plates. Conversion percentages were calculated with consideration of the basal activities (without substrate) and the blanks (without ATP) in comparison to the negative controls (without enzyme).

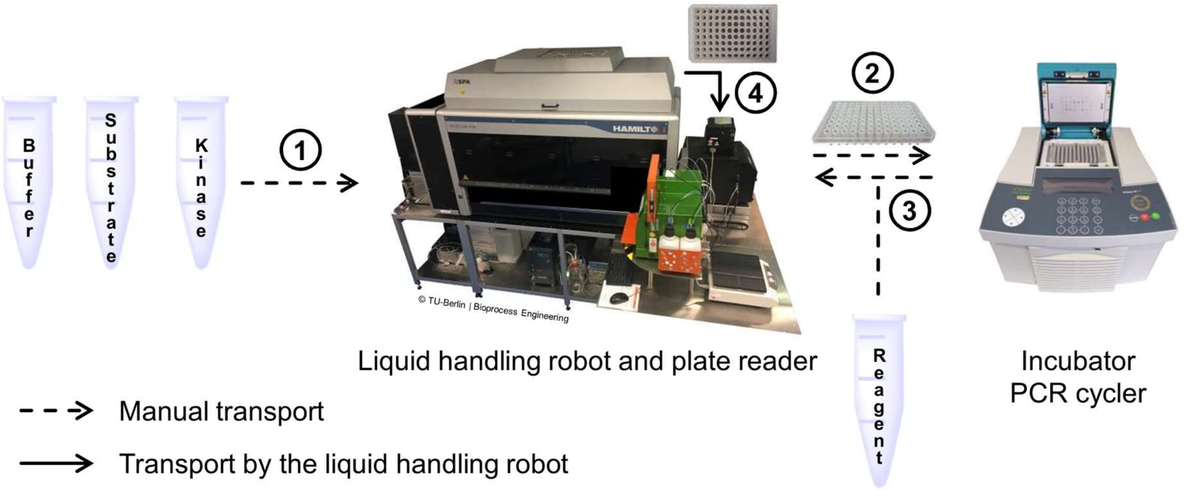

Scheme S1. Semi-automated substrate screening assay for nucleoside kinases. 1- Adding of kinase reaction components to the liquid handling robot and automatic preparation of the kinase reaction. 2- Manual transfer of the kinase reactions to an incubator / PCR cycler for reaction incubation and stop. 3- Manual transfer of the kinase reaction back to the liquid handling robot. Automatic preparation of the luciferase reaction. 4- Automatic transfer of the luciferase reaction to the plate reader. Measurement of the luminescence signal.

Table S3. Assay protocol.

Step	Parameter	Value	Description
1	Deionized water	50[a,b],70[c],100[d] µL	Add to PCR plate
2	5x Reaction buffer	30 µL	Add to PCR plate, final concentration 70 mM Tris HCl [pH 7.6], 10 mM $MgCl_2$, 5 mM DTT, 0 mM[b] or 0.4 mM[a,c,d] ATP
3	Substrate	0[b], 50[a,c,d] µL	Add to PCR plate, 1 mM stocks
4	Enzyme	0[c], 20[a,b,d] µL	Add to PCR plate, prediluted stocks
5	Reaction time	19 h	At 37°C
6	Reaction stop	10 min	At 75°C, lid heat at 85°C
7	Deionized water	80 µl	Add to luminescence assay plate
8	Reaction mixture	10 µl	Transferred from PCR plate to luminescence assay plate
9	Detection mixture	10 µl	Add to luminescence assay plate; Kinase-Glo reagent: prepared according to the manufacturer
10	Incubation time	10 min	At room temperature in the dark in BioTek Synergy Mx Microplate Reader
11	Assay readout	Luminescence	BioTek Synergy Mx Microplate Reader

Step	Notes
	Volumes are for 96w plates.
1	Liquid-handling robot: 8-tip dispense all wells
2	Liquid-handling robot: 1-tip dispense to wells according to pipetting protocol
3	Liquid-handling robot: 1-tip dispense to wells according to pipetting protocol
4	Liquid-handling robot: 1-tip dispense to wells according to pipetting protocol
5	Manual step: plates covered with sealing film and arched auto-sealing lids, transport
6	Manual step: transport
7	Liquid-handling robot: 8-tip dispense to wells according to pipetting protocol
8	Liquid-handling robot: 8-tip dispense to wells according to pipetting protocol
9	Liquid-handling robot: 8-tip dispense to wells according to pipetting protocol
10	Liquid-handling robot: transport to plate reader
11	Plate reader: Luminescence measurement, 1 s and extended dynamic range

[a] kinase reaction, [b] substrate control, [c] negative control, [d] basal activity control (see materials and methods section)

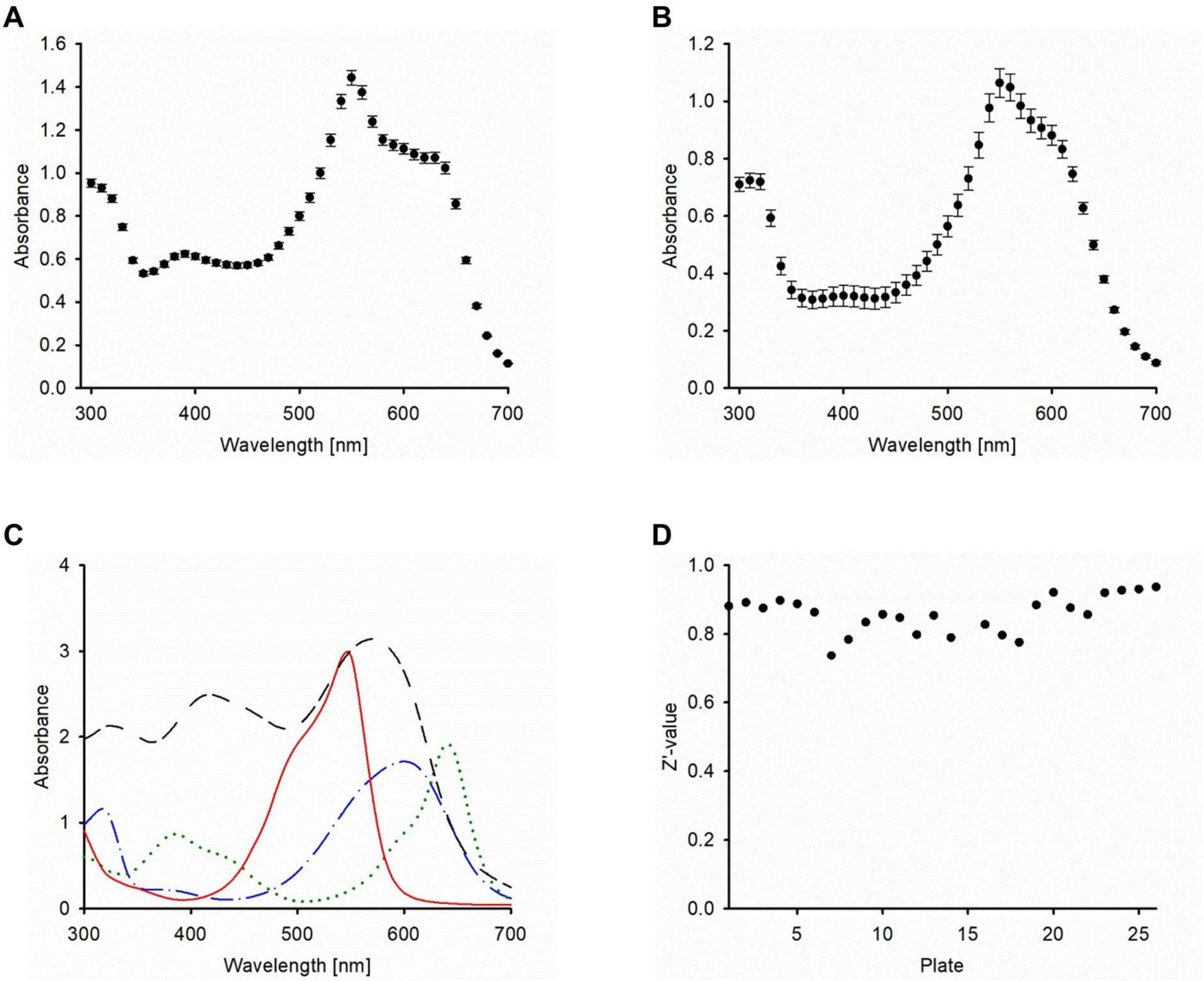

Figure S3. Accuracy of the NK activity assay. For the accuracy tests, the kinase reaction plate and the assay plates were prepared by a liquid-handling robot with colored-solutions. The differences in the absorbance between 300 nm and 700 nm were analyzed. Mean values and standard deviation are indicated. (**A**) The kinase reaction (n = 9) was prepared in a PCR plate by combining blue (water), pink (buffer), green (substrate) and black (enzyme) dye dissolved in water. For the spectral scan, 100 µL were manually transferred to an assay plate. The spectra of the controls are not shown. (**B**) The luminescent assay was prepared twice in assay plates (n = 186) using blue (water), pink (kinase reaction) and black (Kinase-Glo reagent) dye dissolved in water. The spectra were directly measured. (**C**) The spectra of the colored-solutions blue (dashed-dotted), green (dotted), black (dashed) and pink (solid). (**D**) The Z'-values of the luminescent assay for all manually (Plate No. 1-8) and semi-automated (Plate No. 9-26) prepared plates. The Z'-values were calculated with the negative and substrate controls.

Table S4. Activities of four (deoxy)nucleoside kinases with 20 natural (upper part) and modified nucleoside substrates (lower part) were determined using the semi-automated high-throughput NK assay. Conversion percentages [%] were determined in duplicates using the established luciferase assay. Mean values and standard deviations (+/-) are shown.

	***Hs*dCK**	***Hs*AK**	**TK**	***Dm*dNK**
2'-deoxyadenosine	96.2 ± 3.8	18.3 ± 2.2[a]	8.7 ± 8.7	91.8 ± 8.2
2'-deoxycytidine	88.3 ± 1.1	2.3 ± 1.8	25.4 ± 6.1	95.4 ± 4.6
2'-deoxyguanosine	98.1 ± 1.9	0.4 ± 0.4	18.3 ± 13.9	92.9 ± 0.2
adenosine	8.9 ± 0.2	83.0 ± 3.8[a]	0.0 ± 0.0	7.2 ± 0.9
cytidine	97.5 ± 2.5	1.4 ± 1.4	4.0 ± 4.0	70.4 ± 5.8
guanosine	4.6 ± 2.8	0.0 ± 0.0	10.1 ± 7.8	9.9 ± 6.5
thymidine	15.8 ± 0.1	0.1 ± 0.1	88.0 ± 2.5	91.6 ± 0.1
uridine	4.7 ± 0.4	0.5 ± 0.5	35.1 ± 7.5	43.8 ± 0.2
gemcitabine	85.7 ± 1.8	4.3 ± 2.6	9.6 ± 3.0	78.7 ± 1.2
vidarabine	72.0 ± 0.7	4.2 ± 3.9[a]	3.2 ± 1.0	8.0 ± 3.0
ganciclovir	0.0 ± 0.0	0.0 ± 0.0	25.3 ± 1.5	0.0 ± 0.0
1-(2'-deoxy-2'-fluoro-β-D-arabinofuranosyl) uracil	19.6 ± 5.9	0.0 ± 0.0	31.0 ± 0.4	67.2 ± 8.9
5-fluorocytidine	81.8 ± 1.9	5.4 ± 5.4	6.3 ± 4.5	78.8 ± 1.3[c]
5-ethynyl-2'-deoxyuridine	0.0 ± 0.0	0.8 ± 0.8	83.0 ± 17.0	56.4 ± 12.7
lamivudine	46.4 ± 2.0	0.0 ± 0.0	3.2 ± 0.6	23.9 ± 1.6
acyclovir	0.0 ± 0.0	2.2 ± 2.2	28 ± 4.5[b]	0.0 ± 0.0
clofarabine	84.2 ± 5.8	1.8 ± 1.3	8.1 ± 6.8	61.0 ± 7.4
cladribine	80.6 ± 1.0	4.1 ± 3.0	8.4 ± 1.9	67.0 ± 5.6
fludarabine	76.3 ± 2.6	0.0 ± 0.0	5.9 ± 3.7	20.7 ± 1.6
2-fluoroadenosine	46.2 ± 2.7	75.4 ± 3.2[a]	0.8 ± 0.8	19.7 ± 9.8[c]

Reactions consisting of 70 mM Tris [pH 7.6], 5 mM DTT, 10 mM $MgCl_2$, 0.4 mM ATP, 1/3 mM substrate and 0.0002 U enzyme were prepared in a PCR plate to a final volume of 150 µL. After incubation at 37°C for 19 h, reactions were stopped by heat treatment at 75°C for 10 min. Each reaction was analyzed by the luminescent assay in triplicates. Each reaction was prepared as independent duplicates on different PCR plates and was also analyzed on different assay plates. Conversion percentages were calculated with consideration of the basal activities (without substrate) and the blanks (without ATP) in comparison to the negative controls (without enzyme). [a] The reaction buffer contained 50 mM KCl and 0.004 U enzyme. [b] An enzyme concentration of 0.0012 U was applied. [c] An enzyme concentration of 0.004 U was applied.

Table S5. Typical retention times for the HPLC analysis.

Compound	Retention time [min]
adenosine	7.0
adenosine 5'-monophosphate	8.2
adenosine 5'-diphosphate	15.4
adenosine 5'-triphosphate	22.9
2'-deoxyadenosine	7.1
2'-deoxyadenosine 5'-monophosphate	11.0
guanosine	4.0
guanosine 5'-monophosphate	4.6
2'-deoxyguanosine	4.4
2'-deoxyguanosine 5'-monophosphate	6.2
cytidine	2.7
cytidine 5'-monophosphate	3.2
2'-deoxycytidine	2.9
2'-deoxycytidine 5'-monophosphate	3.8
uridine	3.0
uridine 5'-monophosphate	3.6
thymidine	5.5
thymidine 5'-monophosphate	7.4

Samples were analyzed by HPLC-DAD at 260 nm using a reversed-phase column. The flow rate was set to 1 mL min^{-1} at 34°C. The gradient consisted of A (KH_2PO_4/K_2HPO_4: 0.1 M, tetrabutylammonium bisulfate: 8 mM, pH 5.4) and B (70 % A, 30 % MeOH): 0 min – 80 % A, 4 min – 80 % A, 14 min – 40 % A, 35 min - 36.5 % A, 35.5 min – 80 % A and 38 min – 80 % A.

References

[1] A. Brand, L. Allen, M. Altman, M. Hlava, J. Scott, *Learn Publ* **2015**, *28*, 151–155.

[2] K. F. Hellendahl, M. Fehlau, *Zenodo* **2021**, DOI https://doi.org/10.5281/zenodo.5363311.
